# Mechanobiology-informed biomaterial and tissue engineering strategies for influencing skeletal stem and progenitor cell fate

**DOI:** 10.3389/fphys.2023.1220555

**Published:** 2023-07-13

**Authors:** Seth M. Woodbury, W. Benton Swanson, Yuji Mishina

**Affiliations:** ^1^ Yuji Mishina Laboratory, University of Michigan School of Dentistry, Department of Biologic and Materials Science & Prosthodontics, Ann Arbor, MI, United States; ^2^ University of Michigan College of Literature, Science, and Arts, Department of Chemistry, Ann Arbor, MI, United States; ^3^ University of Michigan College of Literature, Science, and Arts, Department of Physics, Ann Arbor, MI, United States

**Keywords:** mechanobiology, biomaterials, tissue engineering, skeletal tissue, stem cell, progenitor cell, microenvironment, dynamic stress

## Abstract

Skeletal stem and progenitor cells (SSPCs) are the multi-potent, self-renewing cell lineages that form the hematopoietic environment and adventitial structures of the skeletal tissues. Skeletal tissues are responsible for a diverse range of physiological functions because of the extensive differentiation potential of SSPCs. The differentiation fates of SSPCs are shaped by the physical properties of their surrounding microenvironment and the mechanical loading forces exerted on them within the skeletal system. In this context, the present review first highlights important biomolecules involved with the mechanobiology of how SSPCs sense and transduce these physical signals. The review then shifts focus towards how the static and dynamic physical properties of microenvironments direct the biological fates of SSPCs, specifically within biomaterial and tissue engineering systems. Biomaterial constructs possess designable, quantifiable physical properties that enable the growth of cells in controlled physical environments both *in-vitro* and *in-vivo*. The utilization of biomaterials in tissue engineering systems provides a valuable platform for controllably directing the fates of SSPCs with physical signals as a tool for mechanobiology investigations and as a template for guiding skeletal tissue regeneration. It is paramount to study this mechanobiology and account for these mechanics-mediated behaviors to develop next-generation tissue engineering therapies that synergistically combine physical and chemical signals to direct cell fate. Ultimately, taking advantage of the evolved mechanobiology of SSPCs with customizable biomaterial constructs presents a powerful method to predictably guide bone and skeletal organ regeneration.

## 1 Introduction

**FIGURE 1:**
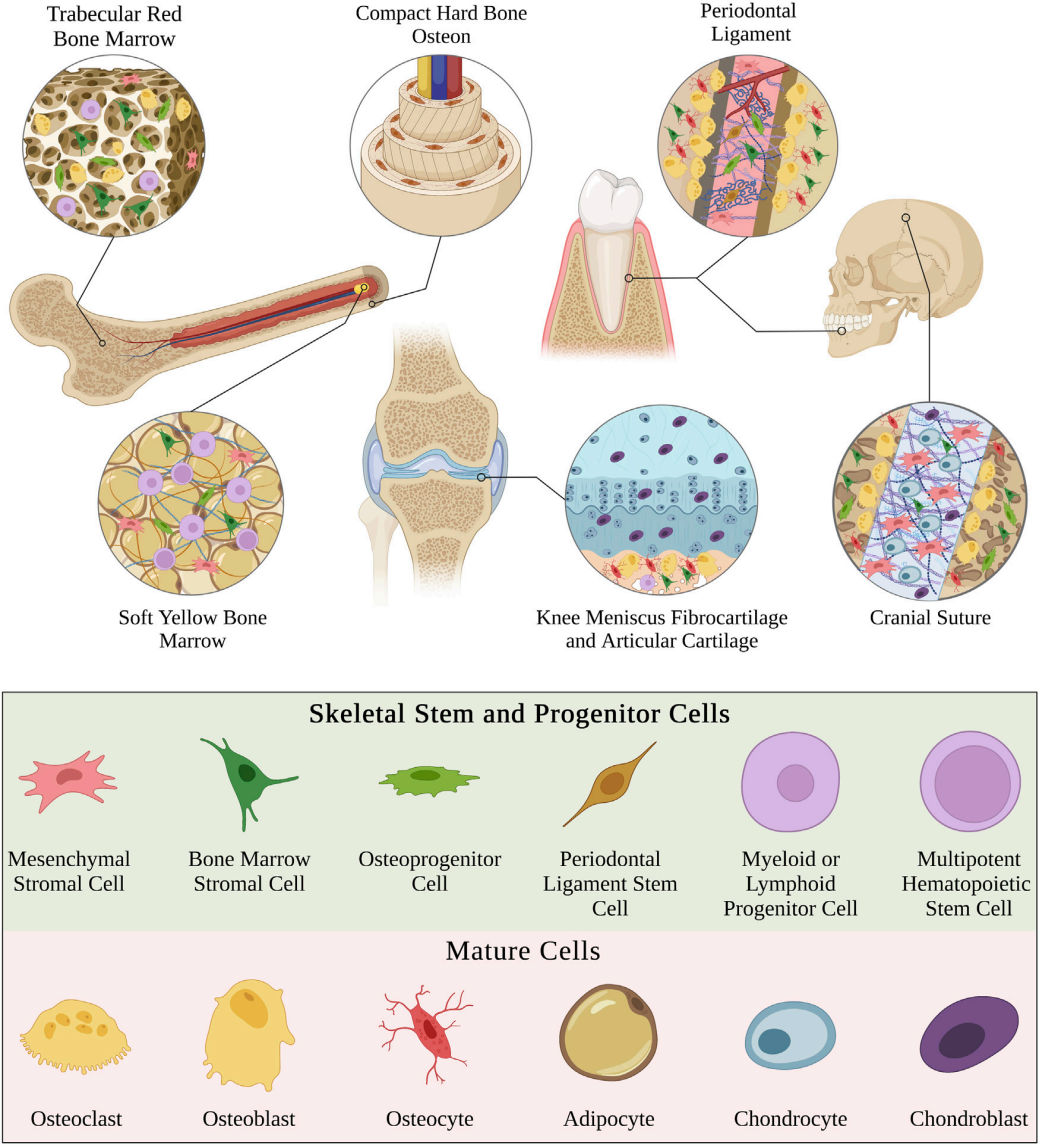
Schematic highlighting diverse examples of different types of skeletal tissues. Note that the physical microenvironment is unique in each type of skeletal tissue and therefore contains different combinations, quantities, and distributions of cell phenotypes. The cell phenotypes displayed throughout this figure are depicted in the bottom legend. This legend holds for the rest of the figures within this manuscript. Figure created with BioRender.com.

Skeletal stem and progenitor cells are the multipotent, self-renewing cell lineages found in the bone marrow stroma that are broadly responsible for the repair, regeneration, and remodeling of skeletal tissue and cartilage (Bianco and Robey, 2015; Matsushita et al., 2020). SSPCs have the potential to differentiate into various cell types such as bone, cartilage, and fat cells (Figure 1). They are also involved in establishing and managing the microvascular network of bone, shaping the hematopoietic environment, and regulating the differentiation of osteoclasts and osteoblasts for bone resorption or deposition, respectively (Bianco and Robey, 2015; Li et al., 2022). Thus, it is paramount to focus on manipulating and strategically utilizing these different behaviors of SSPCs when designing effective techniques to guide bone and skeletal tissue regeneration predictably.

The versatile range of functions that SSPCs possess results from their high sensitivity to the specific chemical and physical microenvironment in which they develop (Kurenkova et al., 2020). The physical microenvironment varies in different types of skeletal tissues and each type hosts unique combinations of cell phenotypes (Figure 1). The physical microenvironment plays an impactful role in the development and remodeling of these tissues by influencing SSPC behavior and differentiation. Moreover, mechanical loading forces imposed on these physical microenvironments also contribute to skeletal tissue remodeling and development. Bone mineral density has been well-demonstrated to increase in the bones of subjects who consistently impose mechanical loading on their appendicular skeletal tissues through weight-bearing exercises (Calbet et al., 1998; Benedetti et al., 2018). Osteocytes were traditionally thought to be the only mechanotransducers in skeletal tissues responsible for this behavior, but modern evidence has proven that SSPCs are also important mechanotransducers that play a significant role in sensing mechanical loading forces and remodeling the tissue (Simmons et al., 2003; Jagodzinski et al., 2004; Li et al., 2004; Scaglione et al., 2008; Grellier et al., 2009). However, the mechanisms by which mechanical forces and physical microenvironments elicit specific SSPC responses have historically remained elusive partially due to the lack of tools to engineer microenvironments with well-controlled physical properties to study SSPC response behaviors (Naqvi and McNamara, 2020). This review aims to highlight important biochemistry mechanisms involved in SSPC mechanotransduction and how tissue engineering strategies have been used to control and study SSPC mechanobiology in different microenvironments.

Mechanobiology is a rapidly emerging field concerned with how cells sense, process, and respond to mechanical information resulting from the extracellular environment (Jansen et al., 2015). It has flourished coinciding with the development and characterization of novel biomaterials and biomaterial construct fabrication methods; engineered biomaterial constructs act as extracellular microenvironments with well-controlled physical properties that allow tissue engineers to study the effects of these properties on the behaviors of different cell types (Hanson et al., 2014; Shafiq et al., 2021). Recent improvements in the understanding of SSPC mechanobiology have lent themselves to inform the next-generation of therapeutic biomaterials and tissue engineering strategies, which account for both physical and chemical cues to guide skeletal tissue and bone regeneration with higher degrees of predictability (Cha et al., 2012; Rahmati et al., 2020). Herein, this review divides biomaterial and tissue engineering physical properties into two main classes (i.e., static or dynamic physical properties) as microenvironment design considerations that guide SSPC behavior and fate.

## 2 Skeletal stem and progenitor cell classification in this review

There is a history of controversy and debate over what exactly constitutes a *bona fide* skeletal stem or progenitor cell due to different reported detection methods, isolation, and functional evaluation (Ambrosi et al., 2019). Bone and skeletal tissues are made up of many heterogeneous stem and progenitor cell lineages that work in conjunction to recruit active hematopoiesis and maintain the integrity of the skeleton (Bianco and Robey, 2015; Serowoky et al., 2020; Li et al., 2022). Several terminologies have been used synonymously in the literature to refer to different sets and subsets of these heterogenous SSPC populations including: ‘multipotent mesenchymal stem/stromal cells’ (MSCs), ‘bone marrow mesenchymal stem/stromal cells’ (BMSCs), and ‘skeletal stem cells’ (SSCs) (Derubeis and Cancedda, 2004; Lindner et al., 2010; Bianco and Robey, 2015; Bhat et al., 2021). Despite being used interchangeably, these terminologies do not mean the same thing and their broad definitions that lack specificity has created an inconsistency in the literature (Ambrosi et al., 2019).

The ISCT minimal criteria for defining the MSC phenotype (Dominici et al., 2006) is an enormously broad definition encompassing cell lineages that have been isolated from many tissues including skeletal, muscular, cardiac, and adipose (Covas et al., 2008; Orbay et al., 2012; Garikipati et al., 2018; Pilato et al., 2018; Pittenger et al., 2019). MSCs isolated from different tissue sources have been experimentally shown to have different transcriptomic profiles and vastly differing differentiation properties (Sacchetti et al., 2016). Importantly, transplanted MSCs isolated from non-skeletal tissues lacked *in-vivo* osteogenic potential and failed to form any histology-proven bone (Sacchetti et al., 2016). Thus, there has been a push towards using the more specific terminology of BMSC or SSC when referring to the subset of MSCs that have been isolated from skeletal tissues, which do demonstrate *in-vivo* osteogenic potential after transplantation and form histology-proven bone (Sacchetti et al., 2007; Bianco et al., 2008; Bianco and Robey, 2015; Sacchetti et al., 2016). Skeletal stem cells defined in this context are an important step toward establishing a definition for a *bona fide* SSPC population, but there are still further caveats within this broad classification. For example, specific markers like Axin2 are expressed in lineages isolated from craniofacial skeletal tissues but are nearly absent in lineages isolated from appendicular skeletal tissues (Maruyama et al., 2016). The different phenotypes among these skeletal stem cell lineages result in different differentiation capacities, functions, and abilities to form hematopoietic and adventitial structures (Ambrosi et al., 2019).

There have been recent evidence-based proposals to enact better criteria for defining a *bona fide* SSPC population and more nomenclature that further specify subsets of SSPC lineages (Ambrosi et al., 2019). Future investigations should be more conscious of how they define the SSPC lineages that they use. This review acknowledges this problem but will broadly define SSPCs as all the heterogeneous stem cell lineages isolated from skeletal tissues that meet the minimum criteria of being multi-potent, self-renewing, and necessary in facilitating the hematopoietic environment or regulating the structural state of bone tissue. Thus, SSPCs in this context include all aforementioned terminologies and others relevant to the regeneration of skeletal tissues since their mechanosensitive mechanisms and microenvironmental response behaviors are generally conserved.

## 3 Relevant biochemistry in skeletal tissue mechanotransduction

Mechanotransduction is at the heart of mechanobiology as it describes the biomolecular mechanisms by which a cell converts a mechanical input into a biochemical signal output dictating a cellular response (Jaalouk and Lammerding, 2009; Martino et al., 2018). SSPCs are particularly mechanosensitive as recent investigations have revealed and elucidated several mechanisms of mechanotransduction that result in their awareness and unique behaviors in different physical microenvironments. The following subsections present a brief introductory overview of the currently understood major mechanotransduction pathways, and their relevance to SSPCs, which are generally conserved in other cell phenotypes as well. These biomolecules and pathways are especially well-studied for SSPCs subject to engineered artificial microenvironments, making them an essential knowledge precursor to designing tissue engineering strategies that guide SSPC proliferation and regeneration.

### 3.1 Focal adhesion kinase

Focal adhesion kinase (FAK) is a non-receptor protein tyrosine kinase found within the cytosol that is referred to as protein-tyrosine kinase-2 (PTK2) in humans (Zachary, 1997; Mitra et al., 2005). FAK is involved in many biochemical pathways controlling cell motility, focal adhesion to the extracellular matrix, cell stiffness, and actin cytoskeleton dynamics (Mitra et al., 2005; Fabry et al., 2011; Yu et al., 2018; Scott et al., 2021). FAK is associated with most of these pathways as a molecular sensor of force that initiates biochemical signals to yield a specific SSPC response. More specifically, FAK acts as a tension sensor between F-actin fibers in the cytoskeleton and the integrins involved in focal adhesions to the extracellular matrix (Bauer et al., 2019; Scott et al., 2021). FAK possesses this ability through its three-domain structure consisting of a kinase active domain sandwiched between a FERM domain, associated with the cell membrane at the focal adhesion, and a FAT domain, associated with the F-actin cytoskeleton fiber (Mitra et al., 2005). The kinase domain is in contact with the FERM domain in the native FAK conformation, blocking the active site of the kinase domain from phosphorylation and subsequent activation (Bauer et al., 2019). Sufficiently high tension between the focal adhesion and F-actin cytoskeleton reversibly unfolds and elongates FAK due to the FERM and FAT domains being pulled in separate directions, exposing the kinase domain. This event causes FAK to become phosphorylated and allows for complexation with Src protein-tyrosine kinase, leading to the subsequent phosphorylation and activation of FAK. In the absence of sufficiently high tension or once the cell relaxes the F-actin cytoskeleton tension in response to FAK activation, FAK will close back into its native low-energy conformation and become inactive (Zhou et al., 2015; Bell and Terentjev, 2017; Bauer et al., 2019). This intricate mechanism is involved in SSPC detection of the stiffness, surface texture, and dimensionality of their environment. Ultimately, the activation of FAK leads to the phosphorylation of many substrates that induce several downstream signaling pathways (Schlaepfer et al., 2004); SSPCs are influenced by the activation state of FAK to craft a unique response to their physical microenvironment (Biggs and Dalby, 2010).

### 3.2 RhoA/ROCK GTPases

GTPases are a class of proteins that hydrolyze GTP to GDP and transduce signals by cycling between GTP-bound active states and GDP-bound inactive states (Etienne-Manneville and Hall, 2002). Rho-family GTPases are a subset of GTPases that are generally involved with cell migration by the remodeling of cellular architecture, which in SSPCs plays an integral role in controlling differentiation and proliferation (Sadok and Marshall, 2014). The most investigated Rho GTPase pathway in SSPC mechanobiology is the Ras-homolog gene family member A (RhoA)/Rho-associated coiled-coil containing protein kinases (ROCK) pathway (Strzelecka-Kiliszek et al., 2017). The RhoA/ROCK pathway transduces signals in response to changes in the F-actin cytoskeletal network as it is affected by F-actin polymerization or depolymerization events, which provides a steady feedback mechanism for SSPCs to regulate their cytoskeleton dynamics and stability (Arnsdorf et al., 2009; Chen and Jacobs, 2013; Martino et al., 2018). The RhoA/ROCK pathway can additionally be activated in response to FAK activation to propagate downstream signals but can also feed into the phosphoryl activation of FAK. In general, the activation of these pathways starts with RhoA activating ROCK to promote the synthesis of stress fibers, which are contractile actin filaments in the cytoskeleton (Tojkander et al., 2012; Scott et al., 2021). Stress fiber formation results in increased cytoskeletal tension that forcibly opens nuclear pores allowing for the nuclear translocation of YAP/TAZ (Section 3.3), which promotes osteogenesis in SSPCs (Elosegui-Artola et al., 2017; Strzelecka-Kiliszek et al., 2017). For the simplicity of this review, it suffices to consider the RhoA/ROCK pathway as a common mediator signal in the mechanotransduction pathways that signal downstream events to occur involved with cell migration, SSPC differentiation fate, and SSPC proliferation via their modulation of cytoskeletal dynamics and interactions with other signaling molecules.

### 3.3 YAP/TAZ

Yes-activated protein (YAP) and transcriptional co-activator with PDZ-binding motif (TAZ) are homologous transcriptional co-activator proteins that influence the expression of genes controlling cell differential fate in SSPCs (Heng et al., 2020). More specifically, YAP/TAZ is heavily implicated in controlling SSPC specification towards adipogenic, osteogenic, or chondrogenic fate through mechanotransduction pathways that promote or inhibit YAP nuclear translocation (i.e., activation) from the cytoplasm or the phosphoryl tagging of YAP for cytosolic degradation (Karystinou et al., 2015; Chang et al., 2017; Pan et al., 2018; Scott et al., 2021). It has been well demonstrated that YAP nuclear translocation and activation inhibit chondrogenesis and adipogenesis, but its role in osteogenesis is conflicting and likely more nuanced. Some evidence suggests that the nuclear translocation of YAP inhibits osteogenesis because of YAP complexation and inhibition of Runx2, a vital transcription factor for osteogenesis (Sen et al., 2015; Chang et al., 2018). However, more recent studies are increasingly associating YAP nuclear translocation with promoting osteogenesis due to YAP binding transcriptional enhancer-associated domain (TEAD) to initiate the transcription of genes related to osteogenesis (Kegelman et al., 2018; Pan et al., 2018; Swanson et al., 2022). The role of YAP in promoting osteogenesis in SSPCs is probably more nuanced than previously thought as activator protein 2a (AP2a) competes with Runx2 to bind YAP in the nucleus, allowing Runx2 to remain free to promote the transcription of genes for osteogenesis (Lin et al., 2018). Furthermore, the AP2a-YAP complexes were shown to interact with the BARX1 promoter to inhibit BARX1 transcription; since BARX1 inhibits osteogenic differentiation, this event helped to promote osteogenesis (Lin et al., 2018). Ultimately, YAP is a complex protein involved in the late stages of the mechanotransduction pathway for SSPCs which favors osteogenic differentiation during nuclear translocation under the right conditions (e.g., AP2a presence), but may inhibit osteogenesis if these conditions are not met (Sen et al., 2015; Kegelman et al., 2018; Lin et al., 2018).

### 3.4 Piezo1/2

The Piezo1/2 ion channels similarly play an important role in the mechanism of mechanotransduction in SSPCs. These transmembrane proteins are composed of numerous transmembrane domains and large extracellular domains, forming a mechanically sensitive complex (Qin et al., 2021). When subjected to mechanical stimuli, such as fluid shear stress or stretching, Piezo1/2 channels experience conformational changes that allow the influx of calcium ions into the cell (Qin et al., 2021). This rise in intracellular calcium triggers a cascade of downstream signaling events, including the activation of various intracellular pathways involved in cell proliferation, differentiation, and gene expression (Fang et al., 2021; Qin et al., 2021).

In the context of SSPCs and bone differentiation, the Piezo1/2 mechanism of mechanotransduction has significant implications. Mechanical forces exerted on skeletal stem cells through physical activity or external loading influence their fate determination and lineage commitment. Activation of Piezo1/2 channels in response to these forces leads to an increase in intracellular calcium levels, initiating a series of molecular events that regulate osteogenic differentiation (Zhou et al., 2020; Ma et al., 2022). This calcium signaling, in conjunction with other signaling pathways, promotes the expression of osteogenic genes and the activation of transcription factors that drive the differentiation of skeletal stem cells into osteoblasts, the bone-forming cells (Li et al., 2019; Zhou et al., 2020; Ma et al., 2022). Consequently, the Piezo1/2 mechanism serves as a critical link between mechanical cues and the regulation of skeletal stem cell behavior, ultimately impacting bone remodeling, adaptation to mechanical stress, and overall skeletal health.

## 4 Static biomaterial strategies

Static biomaterial strategies in this context are defined to be biomaterial systems with fixed physical properties that do not inherently change over most periods of time. Such is often the case with non-active biomaterial constructs, whose properties are determined strictly by their material properties and method of fabrication. For example, titanium dental implants have the fixed material properties of titanium but can be 3D-printed with different structures and surface topologies to affect osseointegration differently (Lee et al., 2022). These subsections explore commonly controlled properties in non-active biomaterial constructs that have been demonstrated to influence the mechanobiology of SSPCs.

### 4.1 Dimensionality

**FIGURE 2:**
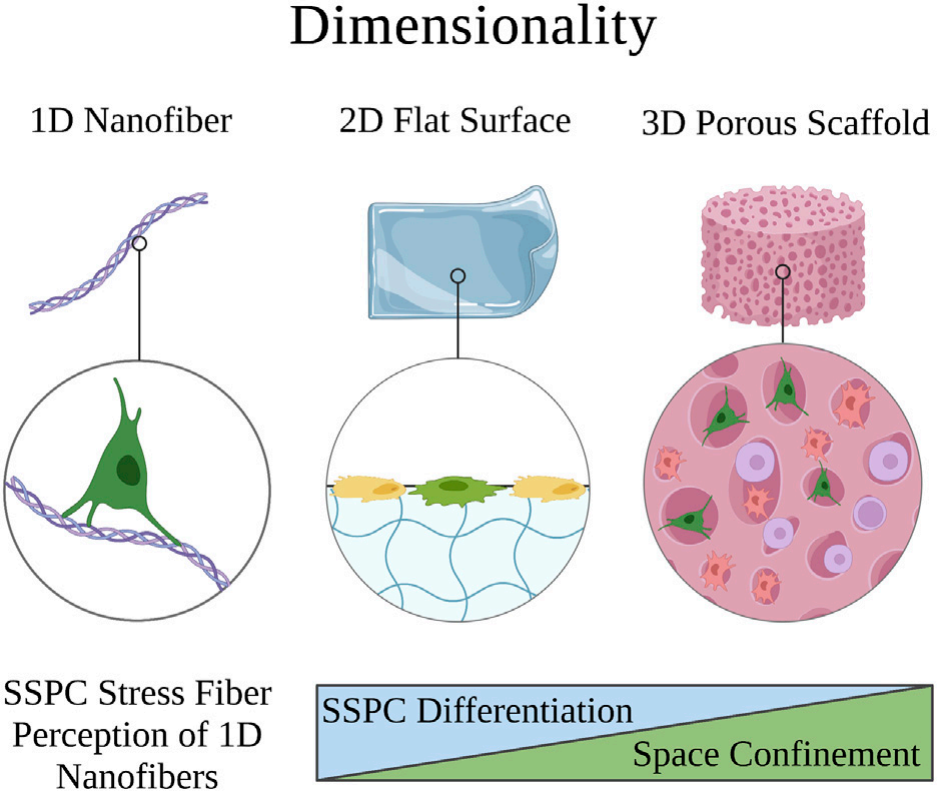
Visual overview of how the perceived microenvironment dimensionality affects SSPC behavior. SSPCs form focal adhesions to surfaces to perceive their dimension. Single nanofibers can be approximated as 1D surfaces and are still perceivable to SSPCs through one or more focal adhesions. 2D microenvironments generally allow SSPCs to spread their shape out into a pancake structure, which promotes differentiation. On the contrary, 3D microenvironments typically confine SSPCs to a more rounded shape, which is associated with maintaining stemness or sometimes promoting adipogenesis or chondrogenesis. A legend depicting the cell phenotypes can be found at the bottom of Figure 1. Figure created with BioRender.com.

Biomaterial constructs are typically two-dimensional (2D; e.g., flat nanofibrous surface) or three-dimensional (3D; e.g., spherical nanofibrous pore) but can also be effectively unidimensional (1D) in the case of single nanofibers (Fang et al., 2022) (Figure 2). The dimensionality of the extracellular environment is sensed by SSPCs by influencing the confinement of their cytoskeletal shape (Robey and Riminucci, 2020; Fang et al., 2022). Specifically, SSPCs spread out on 2D surfaces into ‘pancake’ structures due to a lack of confining static forces in all dimensions (Robey and Riminucci, 2020). This spread, flat shape in SSPCs has been demonstrated to cause increased RhoA activity promoting osteogenesis through the actin-myosin-generated tension in the cytoskeleton (McBeath et al., 2004; Hodge and Ridley, 2016). The increased activity of RhoA increases the activity of ROCK downstream which phosphorylates myosin light-chain kinase and inhibits myosin phosphatase to increase myosin activity (Totsukawa et al., 2000; Scott et al., 2021). This promotes stress fiber formation generating force to open the nuclear pores for YAP nuclear translocation resulting in the promotion of osteogenesis (Tojkander et al., 2012; Elosegui-Artola et al., 2017; Zarka et al., 2022). Conversely, culturing SSPCs in 3D structures allows them to maintain a more rounded, confined shape due to the confining static forces in all dimensions (Remuzzi et al., 2020). This results in the cytoplasmic retention of YAP from the observed decrease in nuclear pore diameter; consequently, there is an upregulation of genes associated with stemness in SSPCs (Heng et al., 2020; Remuzzi et al., 2020). These results ultimately present a fundamental biomaterial strategy to influence SSPCs in a bone tissue engineering context by choosing 2D or 3D biomaterial constructs as the desired platform. Additionally, it suggests that the sensed dimensionality of the microenvironment in different skeletal tissues and regions evolved to purposefully play a role in guiding necessary SSPC shape and fate (Figure 2).

### 4.2 Porosity and pore size

**FIGURE 3:**
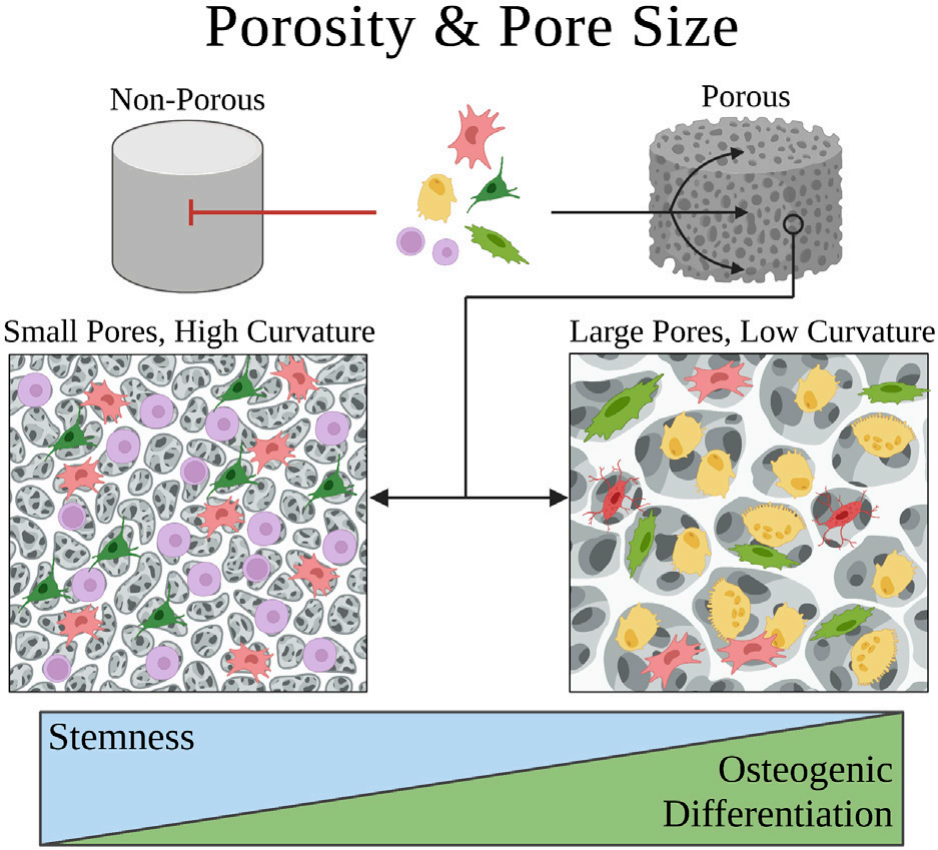
Schematic of how porosity and pore size affect SSPC penetration into a biomaterial and their differential behavior within a biomaterial construct, respectively. SSPCs require a porous biomaterial microenvironment for integration and migration within the construct. The pore size within the biomaterial dictates the principal curvature of the surface, which dictates the degree of confinement imposed on SSPCs. Small pores have high principal curvature and thus impose more shape confinement on SSPCs to promote proliferation and the maintenance of stemness. Conversely, larger pores have lower principal curvatures and impose less shape confinement on SSPCs to promote differentiation and osteogenesis. A legend depicting the cell phenotypes can be found at the bottom of Figure 1. Figure created with BioRender.com.

Porosity is typically associated with 3D biomaterial scaffolds and refers to the average volume of void space (pores) in a given bulk volume of the scaffold. The pores are oftentimes size-controlled with modern porous scaffold fabrication techniques (Loh and Choong, 2013; Chen et al., 2020; Swanson and Ma, 2020). Historically, pores were thought to facilitate the success of tissue engineering constructs by enabling cell and tissue ingrowth rather than fibrous encapsulation (Nunes et al., 1997; Koh and Atala, 2004). More recent evidence suggests that pores may alter the mechanical strain and density of cells, affecting regenerative responses among other potential mechanisms (Swanson et al., 2022). Both the porosity and pore size of biomaterial scaffolds have been demonstrated to affect SSPC behaviors (Figure 3) (Swanson et al., 2022).

SSPCs are most abundantly observed in the bone marrow located in the trabecular bone, which is extremely porous with an average porosity of 79.3%, indicating this evolved design plays a role in the biology of SSPCs (Renders et al., 2007). Scaffolds with higher porosities, given fixed pore size, have been demonstrated to increase SSPC proliferation, migration, and osteogenic differentiation (Aarvold et al., 2013; Dawson et al., 2018). This is hypothesized to be mainly a consequence of increased surface area, which has been demonstrated to lower focal adhesion down-regulating the FAK/RhoA/YAP pathway which promotes gene expression for osteogenesis and osteogenic differentiation (Chang et al., 2018; Dawson et al., 2018). Nonetheless, lower porosity scaffolds do still promote SSPC proliferation compared to non-porous biomaterials, and could perhaps serve useful in applications where maintaining SSPC stemness is crucial such as cranial-suture regeneration, where it is advantageous to maintain a stem cell population rather than purely facilitate osteoblast differentiation (Swanson et al., 2021).

The specific geometric design of pores provides additional design criteria to tune the cell-biomaterial interface (Figure 3). For example, spherical macropores introduce curvature in the biomaterial matrix, where pore size (diameter) influences the curvature experienced by cells in contact with the matrix (Figure 3). It has been demonstrated that the principal curvature of a surface differentially induces cytoskeletal strain and the reorganization of SSPC cytoskeletons (Swanson et al., 2022). The pore size determines the constraint and static force exerted on SSPC cytoskeletons, which was shown to modulate if SSPCs differentiated or maintained stemness via regulation of the YAP/TAZ pathway (Swanson et al., 2022). Sufficiently small pores (<125 µm diameter) with high principal curvature facilitated the upregulation of YAP phosphorylation and its premature degradation in the cytosol to cause maintenance of SSPC stemness within the cell-biomaterial construct. On the contrary, sufficiently large pores (>250 µm diameter) promoted YAP/TAZ complexation and translocation to nuclear targets to induce robust osteogenic differentiation both *in-vivo* and *in-vitro* (Swanson et al., 2022). This is an especially interesting result considering that human trabecular bone has been observed to have a pore size distribution from 50 µm to 850 μm, further suggesting that SSPCs evolved to be mechanosensitive to pore size (Doktor et al., 2011).

### 4.3 Surface topography

**FIGURE 4:**
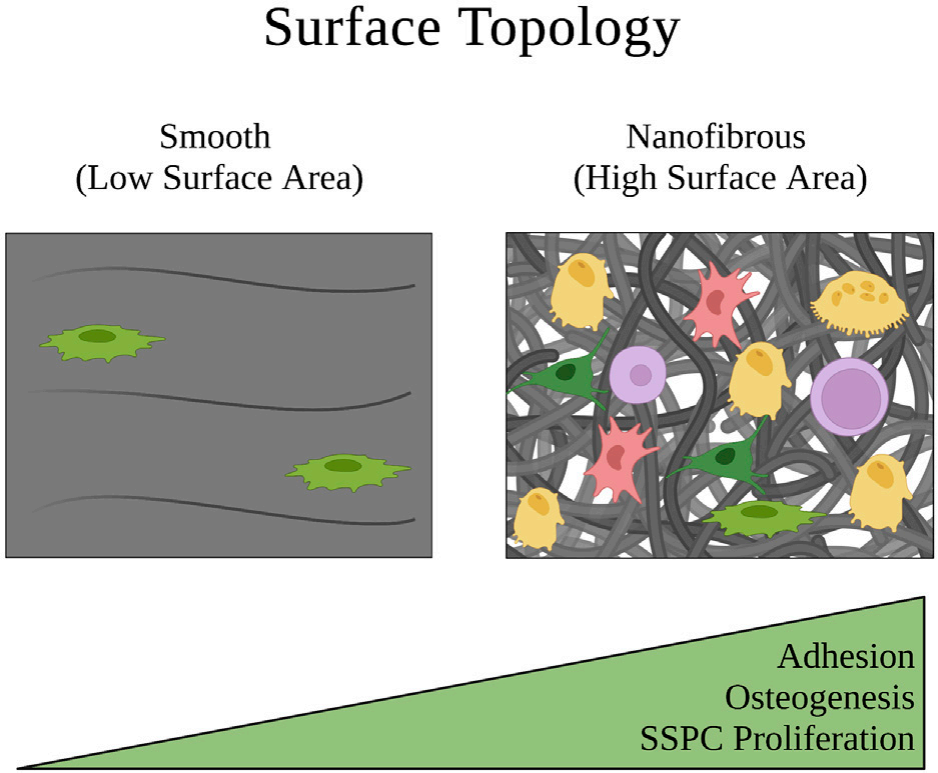
Depiction of how the surface topology of a microenvironment affects SSPC proliferation and behavior. Nanofibers are an ideal surface topology for SSPCs because they maximize surface area. This promotes cell adhesion, proliferation, and generally SSPC differentiation towards an osteogenic fate. A legend depicting the cell phenotypes can be found at the bottom of Figure 1. Figure created with BioRender.com.

Surface topography in a biomaterial context is defined as the interface between the cells and biomaterial, which is often designed to exhibit specific architectures on the micro- and nanometer dimensions or to be a smooth surface (Swanson and Ma, 2020; Vermeulen et al., 2021). SSPCs reside in the trabecular bone, which has a spongy surface topography and interpenetrating extracellular matrix of fibrous collagen type I (Liu and Ma, 2004; McNamara, 2017). Thus, biomaterial strategies that seek to mimic the surface topography of the physical microenvironment in which SSPCs are naturally observed in the bone commonly aim to recreate this nanofibrous surface topography, which has been shown to facilitate cell and protein adhesion compared to smooth matrices (Figure 4) (Vasita and Katti, 2006). Chang et al., 2018 isolated SSPCs from bone marrow and individually cultured cells on either an electrospun, nanofibrous gelatin methacrylate hydrogel (resembles collagen) or a smooth-surface gelatin methacrylate hydrogel. The authors found that SSPCs cultured on nanofibers exhibited higher alkaline phosphatase activity suggesting that nanofibers promote SSPC differentiation and osteogenesis compared to smooth-surface topographies.

Investigation into the mechanism of action led the authors to propose that SSPCs cultured on nanofibers had less focal adhesion causing lower FAK activity and consequently, lower RhoA/ROCK activity (Chang et al., 2018). They further suggested that this downregulation of RhoA/ROCK led to less cellular actin polymerization necessary to translocate YAP from the cytoplasm to the nucleus, ultimately lowering nuclear YAP expression in SSPCs cultured on nanofibers. Because YAP is known to complex with and inhibit Runx2, a vital transcription factor for initiating osteogenic differentiation and osteogenesis, the authors concluded that the decrease in nuclear YAP resulted in increased free Runx2 to initiate the synthesis of alkaline phosphatase causing the enhanced differentiation of SSPCs on nanofibers (Zaidi et al., 2004; Chang et al., 2018). This suggests that the collagen nanofibers found in trabecular bone serve the same effect on the mechanobiology of SSPCs. These results have been extensively replicated with nanofibrous surfaces also created from chitosan, poly-*L*-lactic acid, carbon nanotubules, and other biomaterials where a similar upregulation of Runx2 activity is observed, which promotes alkaline phosphatase and osteocalcin expression as biomarkers of osteogenesis and bone maturation (Ho et al., 2015; Zhang et al., 2016; Das et al., 2018; Yu et al., 2020). There is overwhelming evidence in the literature to suggest that fibrous, and particularly nanofibrous, biomaterials with high surface areas are crucial to SSPC adhesion, proliferation, and differentiation to facilitate osteogenesis (Figure 4); therefore, smooth biomaterial constructs should probably be avoided for skeletal tissue regeneration.

### 4.4 Matrix stiffness

**FIGURE 5:**
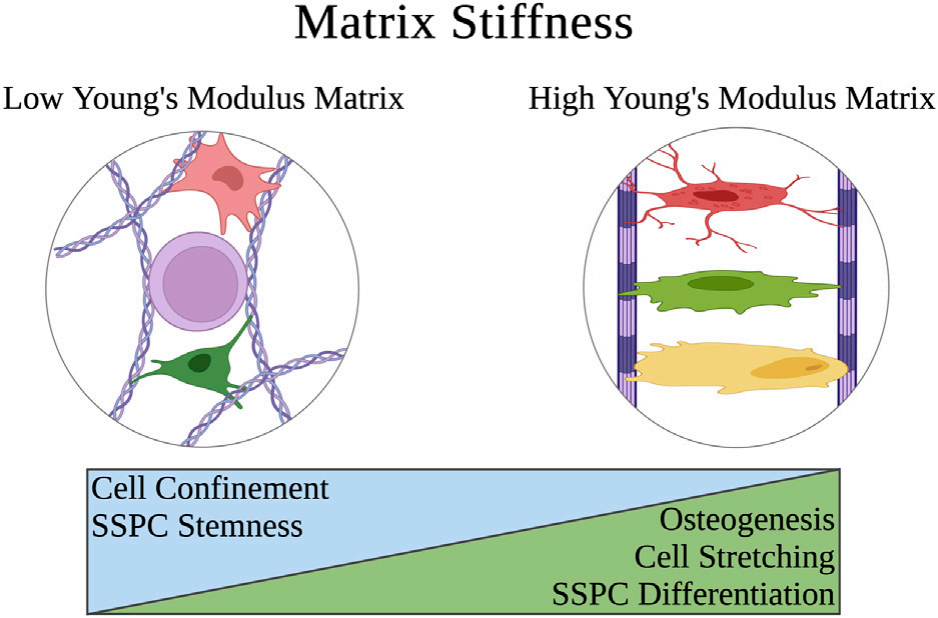
Schematic of how the extracellular matrix stiffness of a physical microenvironment affects SSPC shape confinement and therefore their behavior. Soft matrices have a low Young’s modulus that allows SSPCs to easily deform the matrix when creating cytoskeletal tension on the matrix at the points of stress fiber adhesion. This allows the SSPCs to maintain a rounded, confined shape that is associated with maintaining stemness. On the contrary, rigid matrices with a high Young’s modulus resist deformation when SSPCs exert tension on them which instead causes the SSPC to deform and spread its shape out into a pancake structure. This promotes differentiation and osteogenesis. A legend depicting the cell phenotypes can be found at the bottom of Figure 1. Figure created with BioRender.com.

Extracellular matrix and biomaterial stiffness are typically defined by Young’s Modulus, which describes the magnitude of stress needed to strain a material a given distance. SSPCs and most other stem cells sense the stiffness of their extracellular environment by forming focal adhesions and stress fibers to the surrounding substrates of their microenvironment; this event is followed by constriction of the cellular actin cytoskeleton to generate tension in these adhesion connections and therefore the material of the substrate (Guilak et al., 2009; Burridge and Guilluy, 2016; Smith et al., 2017; 2018; Naqvi and McNamara, 2020). If the material is soft with a low Young’s Modulus, this tension exerted by the cell on the material will cause the material to strain toward the cell, allowing the cell to maintain a more rounded shape (Figure 5). Conversely, if the material is stiff with a high Young’s Modulus, the tension exerted by the cell on the material will strain the cell in the directions of the focal adhesions and stress fibers, causing the cell to stretch out into a flatter pancake shape (Figure 5) (Engler et al., 2006; Sun et al., 2018a; Piroli and Jabbarzadeh, 2018). This is important in the context of influencing SSPC differential fate via the FAK/Rho/YAP mechanotransduction pathway (Dupont et al., 2011; Cai et al., 2021; Li et al., 2021; Scott et al., 2021). Specifically, soft biomaterials have been well demonstrated to guide SSPCs toward adipogenesis, soft to medium stiffness biomaterials promote chondrogenesis, and stiff to rigid biomaterials guide SSPCs toward osteogenesis (Flynn and Woodhouse, 2008; Young et al., 2013; Olivares-Navarrete et al., 2017; Sun et al., 2018b; Smith et al., 2018; Zhang et al., 2018; Roncada et al., 2022; Tang et al., 2022).

Quantitatively, the stiffness of these biomaterials tends to dictate SSPC differential fate in the direction of which natural tissue extracellular matrix Young’s Modulus it most closely resembles, which makes intuitive sense. That is, biomaterials promoting adipogenesis typically have a Young’s Modulus in the range of that of adipose tissue (0.5–2 kPa), biomaterials promoting chondrogenesis typically have a Young’s Modulus in the range of cartilage tissue (500–2000 kPa or 0.5–2 MPa), and biomaterials promoting osteogenesis typically have a Young’s Modulus above these ranges (Rho et al., 1993; Comley and Fleck, 2010; Cox and Erler, 2011; Handorf et al., 2015; Kabir et al., 2021). Ultimately, human SSPCs have evolved to sense the mechanical properties of their extracellular microenvironment as a mechanism to guide proper differentiation in skeletal tissues. Biomaterials that seek to regenerate skeletal tissues can take advantage of this evolved mechanobiology by mimicking their Young’s Moduli (Kozaniti et al., 2022).

## 5 Dynamic biomaterial and tissue engineering strategies

Dynamic strategies in this context are defined to be biomaterial systems with the capacity for inducible changes in their physical properties that effectively change the microenvironment or exert dynamic forces over short periods of time. This requires active biomaterials that can change their physical properties in response to chemical or physical stimuli, such as a piezoelectric material that elongates in an electric field to exert an increased surface tension force along an anchored cell. Additionally, inherently dynamic properties like viscoelasticity are also relevant because they determine how cells physically remodel the microenvironment in time. This can also involve artificial systems that are not necessarily biomaterial in nature but serve the purpose of simulating a dynamic environment (e.g., a dynamic-pressure chamber on a cell culture). Such dynamic strategies are much more limited in the literature but are nonetheless important because they provide an informative basis for how SSPCs respond to acute microenvironment changes and forces, such as those experienced in physical exercise. SSPCs live in a highly dynamic environment and are known to remodel skeletal tissue in response to dynamic forces (Liu et al., 2022); mechanobiological investigations of this phenomenon are necessary to achieve a better understanding of skeletal tissue biology and to design next-generation tissue engineering strategies.

### 5.1 Mechanical loading systems

**FIGURE 6:**
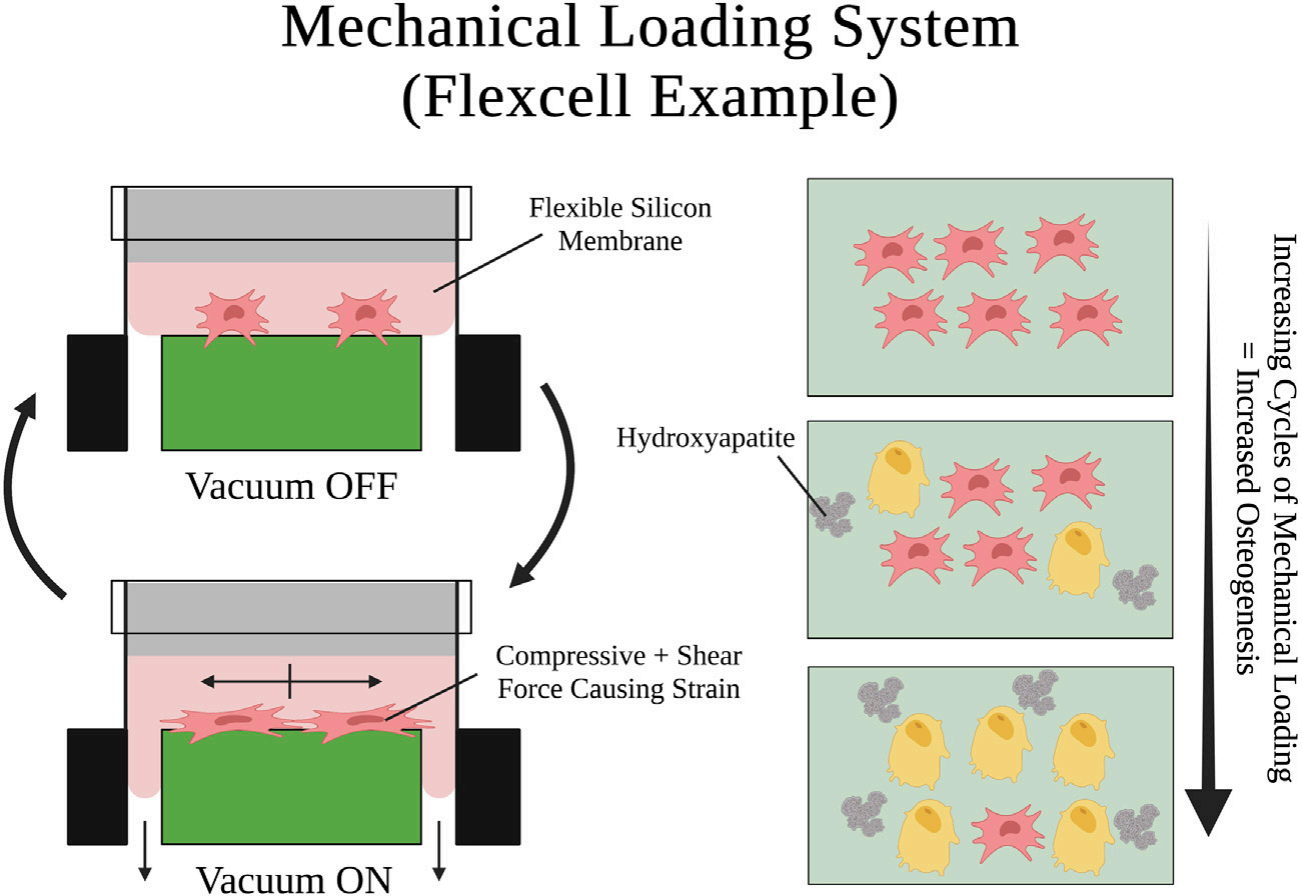
Visual schematic of an example mechanical loading system setup and a mechanical loading cycle using Flexcell^®^. SSPCs are elongated by the shear and compressive tension that the silicon membrane exerts on them when it is pulled by the vacuum. Many repeated cycles of this mechanical loading on SSPCs promotes osteogenic differentiation and mineralization (i.e., hydroxyapatite deposition). A legend depicting the cell phenotypes can be found at the bottom of Figure 1. Figure created with BioRender.com.

Mechanical loading systems are engineered *in-vitro* systems that use the aid of powered machines (e.g., microfluidic injector) or utilities (e.g., vacuum pump) to exert dynamic forces on cultured cells. These devices are oftentimes used to study the behavior of different cells in response to varied magnitudes and exposures to mechanical loading forces, usually compressive or shear forces (Figure 6). Historically, osteocytes were thought to be the dynamic force sensors in the bone because osteons in the hard cortical bone, where the osteocytes reside, receive most of the mechanical loading during physical movement which directly compresses and strains the osteocytes (Weinbaum et al., 1994; Burger and Klein-Nulend, 1999; Taylor et al., 2007; Hart et al., 2017). Although osteocytes are indeed dynamic force sensors that play a role in remodeling skeletal tissue in response to dynamic loading forces, there was a surge of discoveries in the early 2000s demonstrating that SSPCs are also mechanosensitive and aid in the remodeling process with respect to dynamic forces and mechanical loading (Simmons et al., 2003; Jagodzinski et al., 2004; Li et al., 2004; Scaglione et al., 2008; Grellier et al., 2009). This was studied and proved in many cases using mechanical loading systems. Grellier et al., 2009 adapted a parallel-plate culture flow chamber to exert 12 dynes/cm^2^ of laminar fluid shear stress on a flat layer of cultured human-derived SSPCs for 30 and 90 min. They observed an increase in alkaline phosphatase mRNA and connexin43 gene expression, which are associated with osteoblastic lineages and activity (Guillotin et al., 2004). These results suggested that SSPCs are responsive to fluid flow and are specifically driven towards an osteoblastic differential fate in response to shear stress from fluid flow, which thereby promotes osteogenesis. Other studies have adopted similar fluid flow systems and experimental setups with SSPCs to support that mechanical loading in the form of fluid shear stress does induce osteogenic differentiation in SSPCs (Yourek et al., 2010; Sun et al., 2022). SSPC mechanosensitive behavior to fluid shear stress has been demonstrated to result from cell-shape elongation activating the Rho/ROCK/YAP pathway, TRPV4 and Piezo1 mechanosensitive ion channels, and primary cilia, which all collectively transduce the shear stress mechanical signals (Hu et al., 2017; Johnson et al., 2018; Li et al., 2019).

Similarly, Jagodzinski et al., 2004 adopted a cell stretching system to cyclically strain a cultured monolayer of SSPCs longitudinally. They found that a cyclic mechanical strain of 8% applied for 2-h durations three times a day, over 3 days, was able to commit SSPCs to an osteogenic differential fate and acted as a stronger differentiation factor than dexamethasone, a small molecule previously shown to promote SSPC differentiation towards osteoblast fate (Byers et al., 1999). Lohberger et al., 2014 replicated these findings with a similar experiment utilizing the Flexcell FX-5000 Tension System, a mechanical loading system that a computer-controlled vacuum to strain cells adhering to a silicon membrane (Figure 6). For 7 days, they applied continuous cycles consisting of 10 s of mini strain cycles (i.e., five back-to-back cycles of 10% elongation held for 2 s) followed by 30 s of relaxation. They found that the mechanically stimulated groups of SSPCs deposited higher amounts of calcium and alkaline phosphatase into the culture while also increasing their expression of osteogenesis-specific markers (e.g., SPARC, BMP2, SSP1, BGLAP, Col1A1) suggesting that this group of SSPCs was driven towards osteogenic differential fate (Lohberger et al., 2014). Ultimately, the emergence of mechanical loading systems has allowed researchers to investigate the rich mechanobiology that results from the dynamic mechanical environment of bones and movement. There has been overwhelming evidence in the past few decades demonstrating that SSPCs have evolved to be highly sensitive to dynamic mechanical changes in their environment. In most cases, cyclic mechanical loading promotes osteogenesis in SSPCs and guides them to differentiate into osteoblasts (Liu et al., 2022). This is crucial for bone remodeling and serves as a mechanism for bone strengthening and density increases in response to repeated physical activity and exercise.

### 5.2 Active biomaterials

Active biomaterials are a relatively new class of biomaterials that have tunable physicochemical properties in both space and time (Özkale et al., 2021). This review will specifically focus on the subset of active biomaterial constructs that modulate their physical environment in response to external stimuli. These biomaterials are a useful tool for studying how dynamic changes in local mechanical properties influence the mechanobiology of SSPCs in 2D microenvironments (e.g., flat biomaterial sheet) or 3D microenvironments (e.g., porous scaffolds); furthermore, such biomaterial constructs are implantable, which allows for this mechanobiology to be studied with the added complexity of an *in-vivo* microenvironment, unlike the mechanical loading systems (Özkale et al., 2021). One of the most common examples in the literature of active biomaterials that dynamically modulate their physical environment are hydrogels that can stiffen and soften their tensile modulus (Rosales et al., 2017; Lee et al., 2018; Günay et al., 2019). Rosales et al., 2017 created hyaluronic acid-based hydrogels that photodegrade in response to 365 nm light, softening the matrix, and photocrosslinking in response to 400–500 nm light to re-stiffen the matrix. They cultured SSPCs within these hydrogels and found cell area and nuclear YAP/TAZ concentration both positively correlated with increasing hydrogel stiffness, demonstrating that the reversible softening and stiffening of the hydrogel effectively controls the flux of YAP/TAZ between the cell nucleus and cytoplasm. Lee et al., 2018 replicated these findings with SSPCs utilizing a hydrogel made from polyacrylamide and azobenzene, which photoswitches between trans and cis conformations in the presence and absence of blue light, respectively, changing if the matrix is stiff or soft. The SSPC cells and their nuclei were shown to spread more on the stiff matrix than the soft matrix, and the extent of spreading was reversible based on the hydrogel.

More recently, Chen and Lv, 2022 advanced this concept by developing a dynamic hydrogel made from methacrylated gelatin, sodium alginate with calcium carbonate, and D-(+)-gluconic acid δ-lactone that gradually increased in stiffness from 14.63 ± 1.18 kPa to 68.37 ± 4.99 kPa within 7 days (Chen and Lv, 2022). They demonstrated *in-vitro* with SSPCs that this dynamic stiffening promoted osteogenesis more than static hydrogels that were strictly soft or stiff. Moreover, they investigated the regenerative efficacy of this dynamic biomaterial *in-vivo* within calvarial defect models and compared it with soft and stiff static hydrogels. Interestingly, they found that this dynamic stiffening biomaterial significantly enhanced angiogenesis, extracellular matrix remodeling, and bone formation in the critical-sized calvarial defects over 4, 8, and 12 weeks compared to the static soft or stiff hydrogels (Chen and Lv, 2022). The authors hypothesized that the dynamic stiffening environment promoted the expression of extracellular matrix proteins and the secretion of cytokines due to the flattening of SSPCs and subsequent YAP nuclear translocation and activation, committing them to osteogenic differential fates and promoting extracellular matrix remodeling (Lin et al., 2020; Chen and Lv, 2022). Although the exact mechanisms were not investigated, it is obvious from this experiment that SSPCs are sensitive to subtle, gradual changes in the mechanical microenvironment, and this appears to promote their activity with respect to differentiation and participation in remodeling their microenvironment. Ultimately, active biomaterials and dynamic microenvironments may confer unique advantages in bone regeneration because of their ability to activate more mechanisms in SSPCs that favor osteogenesis and other conducive processes for quality bone formation like angiogenesis and extracellular matrix remodeling (Montoya et al., 2021).

### 5.3 Viscoelasticity and stress relaxation

By definition, when stress is applied to biomaterials with viscoelastic properties they undergo an instantaneous, reversible elastic strain followed by a time-dependent, irreversible viscous strain (i.e., plastic deformation) that continues to increase as long as the applied stress force is greater than the biomaterial viscous force (Cohen et al., 1998; Ryeol Choi et al., 2008). Moreover, viscoelastic materials undergo stress relaxation to decrease their tensile stress and internal energy over time when held at a fixed length that puts the body under tensile stress (McHugh et al., 1992). Many biomaterials used in skeletal tissue engineering, namely, hydrogels, have viscoelastic properties (Wu et al., 2022). Chaudhuri et al., 2016 were among the first thoroughly investigate how the viscoelastic stress relaxation rate influences SSPC fate and activity. They fixed the initial elastic modulus at 9 kPa or 17 kPa for all hydrogels and found that hydrogels that were able to relax their internal stress more rapidly (i.e., 1 min relaxation time) caused enhanced YAP nuclear translocation in SSPCs which promoted adipogenesis in the 9 kPa hydrogels, and osteogenesis in the 17 kPa hydrogels.

The authors investigated the mechanism of this behavior and demonstrated that faster matrix stress relaxation promoted SSPC spreading and dynamic shape manipulation to increase YAP nuclear translocation. This occurs because of a positive feedback loop where SSPCs repeat cycles of exerting strain on the hydrogel followed by stress relaxation (Chaudhuri et al., 2016). Each cycle of stress relaxation relieves the tension initially exerted by the cytoskeleton. This change in the cytoskeleton dynamics is transduced by the actomyosin and Rho signaling pathways to create more focal adhesions around the new plastic-deformed biomaterial by the pre-existing focal adhesions. The SSPCs repeat the cycles and continue to reinforce their focal adhesions which stretches their shape and nuclear pores for YAP nuclear translocation (Chaudhuri et al., 2016). Other studies have replicated these results *in-vivo*, *in-vitro*, and with SSPC spheroids arriving at the common conclusion that fast stress relaxing viscoelastic biomaterials promote SSPC proliferation, migration, differential fate towards osteogenesis, and fusion with surrounding tissues *in-vivo*, and mature bone formation (Darnell et al., 2017; Wu et al., 2022). The ability for the extracellular matrix to be plastically deformed and remodeled by SSPCs in viscoelastic biomaterials because of their ability to dissipate internal stress imposed by cell pulling forces, leading to plastic deformations, as opposed to purely elastic biomaterials, appears to be highly conducive to osteogenesis and bone regeneration. This is likely because such biomaterials imitate the malleable, fast-relaxing viscoelastic properties of type I collagen that lend themselves to dynamic physicochemical remodeling by SSPCs (Yamashita et al., 2002). Ultimately, the field of tissue engineering can benefit by accounting for these dynamic properties like viscoelasticity and stress relaxation.

### 5.4 Elasticity

It is worth mentioning that highly elastic biomaterials, such as poly(ester)urethane or poly(lactide-co-caprolactone), have been demonstrated to promote chondrogenesis with SSPCs (Jung et al., 2009; Camarero-Espinosa et al., 2020). Cartilage tissues exhibit viscoelastic properties, but they are best described as mostly elastic because they store significant amounts of elastic energy and do not stress-relax very rapidly. These properties primarily derive from type II collagen, which makes up a significant portion of most cartilage tissues, especially articular cartilage (Silver et al., 2002). Thus, SSPCs and chondrocytes in cartilage cannot physically remodel their microenvironment as easily by exerting cycles of stain followed by microenvironment stress-relaxation, as is possible in bone with type I collagen. This clearly plays a role in the mechanobiology driving SSPCs toward chondrogenesis in elastic microenvironments, but the exact mechanisms driving this chondrogenic fate have not been thoroughly investigated or well understood (Jung et al., 2009; Somoza et al., 2014; Camarero-Espinosa et al., 2020). Nonetheless, biomaterials with static and dynamic properties resembling native skeletal tissues drive SSPC differential fate toward the specific progenitors and cell types present in those tissues, as observed with viscoelastic biomaterials promoting osteogenesis and elastic biomaterials promoting chondrogenesis (Jung et al., 2009; Darnell et al., 2017; Camarero-Espinosa et al., 2020; Wu et al., 2022).

### 5.5 Mechanotransduction-growth factor interactions

Growth factors, such as transforming growth factor-beta (TGF-β) and platelet-derived growth factor (PDGF), facilitate cell proliferation, differentiation, and extracellular matrix synthesis (Caplan and Correa, 2011; Li et al., 2014; Wu et al., 2016; Kwak and Lee, 2019). Meanwhile, mechanotransduction signaling involves the conversion of mechanical forces into biochemical signals, triggering cellular responses and modulating tissue remodeling. By integrating these two approaches, the synergistic effects of growth factors and mechanotransduction signaling can be harnessed to enhance cell behavior, promote tissue maturation, and optimize the mechanical properties of engineered tissues (Dang et al., 2018). This integration holds great promise for advancing tissue engineering strategies, allowing the creation of functional and biomimetic tissues for various regenerative medicine applications, and is an area of active investigation where matrix-derived cues and soluble factors synergistically influence differentiation trajectories of SSPCs (Kusuma et al., 2017).

The differentiation status of SSPCs has been shown to influence their paracrine activity with distinct changes occurring during osteogenic, chondrogenic, and adipogenic lineage commitment (Choi et al., 2010). In fact, conditioned media from osteogenic SSPCs cultures, both mechanically induced and chemically induced, enhances the differentiation process in recipient cells *in-vitro* (Frith et al., 2013). In particular, mechanical loading has been shown to increase angiogenic paracrine factors within various SSPC populations, namely, MMP2, TGF, and FGF (Kasper et al., 2007). Other studies have similarly shown that limiting cell spreading (and cytoskeletal architecture) depleted VEGF, IGF, and EGF secretion (Kilian et al., 2010; Abdeen et al., 2014). These matrix-derived cues within the engineered cell microenvironment further tune the regenerative trajectory towards specific, predictable tissue fates.

## 6 Discussion

Skeletal tissues contain heterogeneous physical microenvironments that are sensed by SSPCs to guide their differentiation fate (Miller et al., 2015; Chen et al., 2020). Understanding how the static and dynamic physical properties of native and biomaterial microenvironments are transduced by SSPCs is an important step toward developing more predictable, quality regenerative therapies for skeletal tissues. Certain physical properties for biomaterial designs aimed to engineer skeletal tissues appear to have a universally desirable option; for example, nanofibrous surface topologies of the SSPC microenvironment universally promote adhesion and proliferation (Nemati et al., 2019; Yu et al., 2020). In contrast, flat surface topologies are not as adhesive and limit cell proliferation and migration in general. However, most properties and design considerations lie on a spectrum where the ideal design depends on the goal of the tissue outcome. Matrix stiffness is an example of this because it lies on a continuous numerical spectrum where soft substrates promote adipogenesis, medium-stiffness substrates promote chondrogenesis, and stiff substrates promote osteogenesis (Park et al., 2011; Assis-Ribas et al., 2018). Similarly, there is good evidence that microenvironment viscoelasticity is a mechanical cue for determining whether SSPCs commit to a chondrogenic fate, which occurs in elastic materials, or an osteogenic fate, which occurs in rapid stress-relaxing viscoelastic materials (Chaudhuri et al., 2016; Begum et al., 2020).

Several factors such as pore size and perceived microenvironment dimensionality control whether SSPCs maintain a round shape and their stemness, or if they flatten out and differentiate (Clause et al., 2010). In fact, most microenvironment physical factors influence SSPC shape and thus it is important to consider the balance between these microenvironmental properties when designing biomaterials and tissue engineering strategies for skeletal tissue regeneration. Oftentimes these mechanical properties may redundantly command SSPC shape and mechanotransduction. For example, macroporous trabecular bone with a relatively stiff but fast-relaxing viscoelastic, nanofibrous extracellular matrix promotes SSPC spreading and therefore differentiation and osteogenesis (Oftadeh et al., 2015). This can be further enhanced with mechanical loading in the form of weight-bearing exercise, which further strains SSPCs through compressive force and shear fluid flow throughout the porous bone to stimulate higher rates of osteogenesis through similar redundant cell-shape spreading mechanobiology pathways (Zernicke et al., 2006). Ultimately, these physical cues work in combination with each other, as native skeletal tissues have carefully evolved to reproducibly guide the SSPCs toward their desired fates with controlled combinations of physical and chemical cues.

From an engineering perspective, it is interesting to consider the cases where different physical microenvironmental cues may conflict with each other in determining skeletal tissue outcomes. For example, consider a small-pore scaffold made from a purely elastic biomaterial with a stiff tensile modulus. The small pores would be predicted to encourage the SSPCs to maintain a rounded shape, promoting stemness, while the elastic properties of the biomaterial may also contribute to a generally more rounded SSPC shape but usually favor chondrogenesis (Begum et al., 2020; Swanson et al., 2022). In contradiction, the stiff tensile modulus tends to cause SSPCs to spread their shape which promotes osteogenic fate. But to what degree will the SSPCs spread out? Will it maintain stemness or be driven to osteogenic or chondrogenic fates? Perhaps the outcome will change if the biomaterial can actively stiffen and soften or impose artificial mechanical loading on the SSPCs.

**FIGURE 7:**
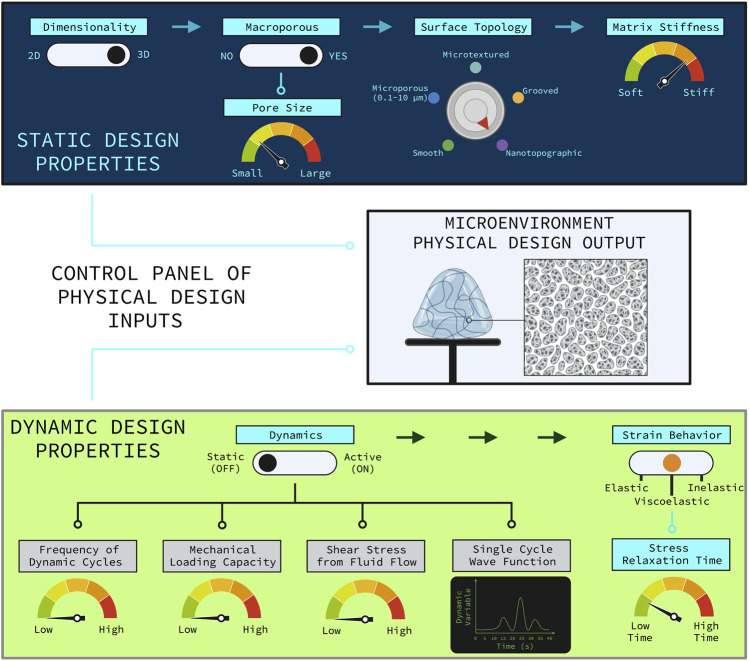
Control panel representation of the relevant physical design parameters for designing a biomaterial construct for tissue engineering. The control panel is divided into the static physical design properties (top, blue panel) and the dynamic physical design properties (bottom, green panel). The rational combination of independent and dependent design variables as illustrated allow for various combinations of unique biomaterial environments to guide SSPC trajectories in predictable ways. Design variable names are colored blue if they are applicable or grey if they are not applicable. The independent design variables are horizontally distributed with the arrows at the top of each panel. Design variables that are conditionally dependent on the choice of an independent variable are connected by nodes to the independent variable that it is conditionally dependent on. If an independent variable choice makes the conditionally dependent variable(s) applicable then the node(s) turn light blue; otherwise, the nodes are black meaning that the conditionally dependent variable(s) are not applicable. The hypothetical biomaterial physical microenvironment depicted in the middle box was designed with this control panel to be non-active with fast-relaxing viscoelastic properties, three-dimensional, macroporous with smaller sized macropores, nanotopographic (i.e., nanofibrous), and relatively stiff. Note that utilizing a non-active biomaterial significantly hinders the potential to customize the biomaterial, which generally sacrifices a degree of regenerative outcome predictability and efficacy. It is also important to note that the many static and dynamic physical properties of a microenvironment act in combination to holistically influence SSPC behavior and fate. Figure created with BioRender.com.

These are important questions to investigate which will yield a greater understanding of the underlying mechanobiology of how SSPCs sense and respond to complex combinations of physical cues in their microenvironment and which properties of the microenvironment are more important in dictating SSPC differential fate. Understanding how combinations of physical microenvironment properties work synergistically to drive SSPC behavior will inform the next-generation of optimized biomaterial niches and tissue engineering strategies for regenerating skeletal tissues with higher degrees of quality and predictability. This ultimately provides tissue engineers with a control panel of physical property design customizations that can be used to control SSPC differential fate and behavior, allowing for the possibility of precisely and reproducibly engineering specific skeletal tissues that are of interest to the engineer (e.g., articular cartilage, trabecular bone) (Figure 7). Note that some design variables are independent and always applicable (e.g., dimensionality, surface topology) while other design variables are conditionally applicable depending on the choice of an independent variable (Figure 7). For example, the depicted biomaterial designed by the control panel is macroporous and thus requires the pore size to be specified (Figure 7). If the biomaterial was not macroporous then the pore size variable would not be applicable.

Among the important physical strategies for producing desirable SSPC regenerative outcomes, the studies presented on active biomaterials and mechanical loading systems suggest that dynamic physical environments and mechanical loading are universally advantageous strategies for promoting angiogenesis, migration, proliferation, and differentiation (Rosales et al., 2017; Lee et al., 2018; Günay et al., 2019; Chen and Lv, 2022). This is a relatively new area of biomaterials, tissue engineering, and mechanobiology that is ripe for exploration. Mechanisms of mechanotransduction for these dynamic properties are still under investigation especially as new tools and methods are developed for studying mechanobiology (Mohammed et al., 2019). With respect to tissue engineering, there is a lack of methods to controllably impose reversible, specific, known mechanical loading forces on 3D skeletal tissue and SSPC microenvironments *in-vivo*. An active biomaterial scaffold would be ideal for this task because they are 3D and usually implantable, but few constructs have been synthesized that can reversibly impose mechanical loading *in-vivo* to external stimuli on demand. Developing an active biomaterial tool to quantitatively investigate dynamic mechanobiology for SSPCs in a complex *in-vivo* environment would help quantify the desirable dynamic properties and mechanical loading forces necessary for a healthy skeletal microenvironment. It would be a useful tool to investigate how fluid flow through porous environments, in response to mechanical compression, mediates nutrient exchange and influences SSPC fate with shear stress *in-vivo*. Finally, it would help inform a new class of regenerative engineering strategies that utilize dynamics and statics to modulate SSPC mechanobiology for tissue repair and regeneration.

Equally crucial to developing new biomaterial constructs and tools is paramount mapping out the intricate molecular mechanisms by which SSPC mechanotransduction occurs in both static and dynamic microenvironments. Significant progress has been made on this in the past two decades with the emergence of novel tools and methods in molecular biology for studying proteomics and gene expression, but there is still ambiguity with the role of even the most well-studied central biomolecules in mechanotransduction. As previously mentioned, there is a history of controversy and debate as to whether YAP nuclear translocation promotes or inhibits osteogenesis (Kegelman et al., 2018; Pan et al., 2018). Most recent studies have arrived at the consensus that YAP nuclear translocation does generally promote osteogenesis, but it is unknown why many of the groups investigating nanofibrous surface topologies found that osteogenesis was promoted from a lack of YAP nuclear translocation, even though cell-shape spreading was observed (Zaidi et al., 2004; Chang et al., 2018). This dilemma suggests that other mechanotransduction pathways feed into stimulating osteogenesis not necessarily reliant on YAP. For example, Chaudhuri et al., 2016 found the same range of nuclear YAP levels in SSPCs cultured in their 9 kPa and 17 kPa viscoelastic hydrogels but observed the SSPCs differentiate towards adipogenesis in the soft hydrogel and osteogenesis in the stiffer hydrogel. Thus, nuclear YAP levels were surprisingly decoupled from SSPC fate, even though it is known to likely stimulate osteogenesis and inhibit adipogenesis (Chaudhuri et al., 2016). This demonstrates that YAP mechanotransduction and signaling are still not completely understood, and it probably plays a much more non-canonical and nuanced role in SSPC differential fate. Additionally, nuclear YAP immunohistostaining is not sufficient by itself to evaluate how SSPCs respond to physical cues or if this is what triggers their differentiation. Future investigations should look for other mechanotransduction pathways and systematically probe the behavior of known biomolecules that play a role in SSPC mechanobiology by studying their proteomics and spatiotemporal omics when SSPCs are exposed to different physical environments.

## 7 Conclusion

Next-generation tissue engineering strategies require an understanding of the underlying mechanobiology by which biomaterial and microenvironment physical properties influence SSPC behavior. Culturing SSPCs in artificial biomaterial microenvironments with known physical properties, both *in-vitro* and *in-vivo*, and evaluating their phenotypic outcomes has shed some perspective on how native skeletal tissues and biological materials reproducibly guide SSPC fate. Furthermore, this has led to an increased understanding of the molecular mechanisms by which skeletal organs and cells transduce mechanical stimuli. These mechanical stimuli derive from the physical properties of the microenvironment, which can be broadly categorized as static or dynamic. These properties all work in conjunction to control SSPC behavior and therefore it is paramount to consider how different combinations of all the static and dynamic physical properties in a microenvironment dictate SSPC outcomes when engineering novel strategies to regenerate skeletal tissues with precision and predictability. There are still many questions and challenges ahead in this interdisciplinary collaboration to understand and engineer the SSPC physicochemical microenvironment, but significant progress has been established in just the past decade and the field only continues to grow.
